# Multiple signals modulate the activity of the complex sensor kinase TodS

**DOI:** 10.1111/1751-7915.12142

**Published:** 2014-07-01

**Authors:** Hortencia Silva-Jiménez, Álvaro Ortega, Cristina García-Fontana, Juan Luis Ramos, Tino Krell

**Affiliations:** 1 Department of Environmental Protection, Consejo Superior de Investigaciones Científicas, Estación Experimental del ZaidínC/ Prof. Albareda 1, Granada, 18008, Spain; 2 Abengoa Research, Campus Palmas AltasC/Energía Solar n° 1, Seville, 41014, Spain

## Abstract

The reason for the existence of complex sensor kinases is little understood but thought to lie in the capacity to respond to multiple signals. The complex, seven-domain sensor kinase TodS controls in concert with the TodT response regulator the expression of the toluene dioxygenase pathway in *Pseudomonas putida* F1 and DOT-T1E. We have previously shown that some aromatic hydrocarbons stimulate TodS activity whereas others behave as antagonists. We show here that TodS responds in addition to the oxidative agent menadione. Menadione but no other oxidative agent tested inhibited TodS activity *in vitro* and reduced P*_todX_* expression *in vivo*. The menadione signal is incorporated by a cysteine-dependent mechanism. The mutation of the sole conserved cysteine of TodS (C320) rendered the protein insensitive to menadione. We evaluated the mutual opposing effects of toluene and menadione on TodS autophosphorylation. In the presence of toluene, menadione reduced TodS activity whereas toluene did not stimulate activity in the presence of menadione. It was shown by others that menadione increases expression of glucose metabolism genes. The opposing effects of menadione on glucose and toluene metabolism may be partially responsible for the interwoven regulation of both catabolic pathways. This work provides mechanistic detail on how complex sensor kinases integrate different types of signal molecules.

## Introduction

Two-component systems (TCSs) are a major bacterial signal transduction mechanism and regulate virtually all types of cellular processes (Galperin, 2005; Mascher *et al*., 2006; Krell *et al*., 2010). The basic components of a TCS are a sensor kinase and a response regulator. There is an important diversity in the architecture of both proteins, which has led to the differentiation between prototypal and more complex, hybrid TCS. Typically, a prototypal sensor kinase is composed of a sensor domain and a transmitter module. Signal binding to the sensor domain modulates transmitter module autokinase activity, which in turn modulates the transphosphorylation activity to the response regulator receiver domain. However, a large number of TCSs possess a more complex architecture. There are sensor kinases that possess multiple copies of the same domain type or harbour additional domains like histidine containing phosphotransfer domains (Krell *et al*., 2010). This complexity in architecture is frequently reflected in a more complex mechanism as many of these TCS employ a His_1_-Asp_1_-His_2_-Asp_2_ phosphorelay instead of a simple His-Asp phosphoryl-transfer of the prototypal system (Pena-Sandoval *et al*., 2005; Zhang and Shi, 2005; Cock and Whitworth, 2007; Busch *et al*., 2009). The physiological relevance of this complexity is not clear, but it has been suggested that it may lie in their capacity to integrate different signals (Burbulys *et al*., 1991; Stephenson and Hoch, 2001; Cotter and Jones, 2003), causing ultimately a fine-tuning of the response. However, there is only a limited knowledge on how multiple signals are integrated into the signalling cascades of complex TCS.

**Fig 1:**
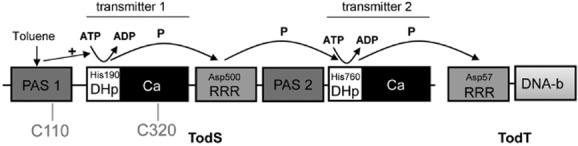
Schematic representation of the domain organization and mode of action of the TodS/TodT TCS. The phosphorylgroup-accepting residues and the phosphorelay are indicated as established by Busch and colleagues (2009). PAS, Per-Arnt-Sim-type sensor domain; DHp, dimerization/histidine phosphotransfer domain; Ca, catalytic domain; RRR, response regulator receiver domain; DNA-b, DNA-binding domain; P, phosphorylgroup.

We have addressed this question using the complex TodS/TodT TCS of *Pseudomonas putida* DOT-T1E, which was shown to modulate the expression from promoter P*_todX_* that controls the genes of the toluene dioxygenase pathway (TOD) for the metabolization of benzene, toluene and ethylbenzene (Zylstra and Gibson, 1989; Lau *et al*., 1997; Mosqueda *et al*., 1999). The 108 kDa sensor kinase TodS is composed of two transmitter modules each comprising a dimerization/histidine phosphotransfer domain and a catalytic domain (Fig. 1). Each transmitter module is preceded by a Per-Arnt-Sim (PAS) domain, and a receiver domain is found in the centre of the TodS sequence. We have shown previously that TOD pathway expression is induced by a range of aromatic compounds like toluene (Lacal *et al*., 2006) and have demonstrated that these effectors bind to the N-terminal PAS domain, increasing the activity of the N-terminal autokinase module (Lacal *et al*., 2006; Busch *et al*., 2009). In addition to these agonists, we have identified structurally very similar compounds (o-xylene, for example) that also bind to the N-terminal PAS domain, but do not stimulate TodS autophosphorylation (Busch *et al*., 2007). The presence of these compounds (termed antagonists) was found to reduce the magnitude of agonist-mediated upregulation (Busch *et al*., 2007). The TodS phosphorylation state is thus controlled by the concerted action of agonists and antagonists that compete for the same site at TodS. Similar observations were made for the homologous system TmoS/TmoT that controls the toluene-4-monooxygenase degradation pathway in *Pseudomonas mendocina* (Silva-Jimenez *et al*., 2012). We were also able to show that TodS operates by a His_1_-Asp_1_-His_2_-Asp_2_ phosphorelay mechanism (Busch *et al*., 2007; 2009) (Fig. 1). TodS lacks transmembrane regions and can be obtained as soluble and active protein (Lacal *et al*., 2006).

Strains of *P. putida* are characterized by an extraordinary metabolic diversity (Timmis, 2002) and resistance to different stressors that makes them well-suited candidates for biodegradation purposes (Ramos *et al*., 2002). However, frequently bioremediation processes developed did not show the necessary efficiency to be used on larger scales (Cases and de Lorenzo, 2005). One of the current limitations of biodegradation is the insufficient gene expression of degradation pathways under *in situ* conditions, which enhances the need for further research.

We wanted to establish whether other signal molecules modulate TodS activity. A number of other sensor kinases are sensitive to the redox potential. Several mechanisms have evolved to integrate such signals that are based on cofactor containing sensor domains, metal-sulfur clusters or the modification of cysteine residues (Bauer *et al*., 1999; Zheng and Storz, 2000; Antelmann and Helmann, 2011). It has been shown that the activity of several other complex sensor kinases like ArcB, EvgS and BvgS (Georgellis *et al*., 2001; Bock and Gross, 2002) is modulated by quinones via a cysteine dependent mechanism (Malpica *et al*., 2004). Because the quinone concentration depends on the redox state of the cell, this mechanisms permits thus to integrate redox signals. We show here that, apart from aromatic hydrocarbons, TodS activity is modulated by menadione via a cysteine dependent mechanism. Menadione (2-methyl-l,4-naphthoquinone, vitamin K3) and other quinones are redox-active compounds synthesized by bacteria (Nowicka and Kruk, 2010). Because of their hydrophobicity, they integrate into the membrane and, depending on the redox state of the cell, form an equilibrium with their quinol derivatives. The ability of reversible reduction makes quinones ideal candidates for their function as hydrogen shuttles between different protein complexes of biological membranes (Nowicka and Kruk, 2010). Menadione was found to modulate gene expression in bacteria (Kohler *et al*., 2008), and a number of studies show a direct effect of menadione on the activity of several transcriptional regulators as exemplified by ArcBA (Georgellis *et al*., 2001; Bekker *et al*., 2010; Alvarez *et al*., 2013) and MarR of *Escherichia coli* (Alekshun and Levy, 1999), SoxR and GapR of *Pseudomonas aeruginosa* (Singh *et al*., 2013; Deng *et al*., 2014) or HexR of *P. putida* (Kim *et al*., 2008). Our results are discussed in the context of a report showing that menadione stimulated glucose metabolism in *P. putida*.

## Results

### Reducing conditions are essential for TodS activity

**Fig 2:**
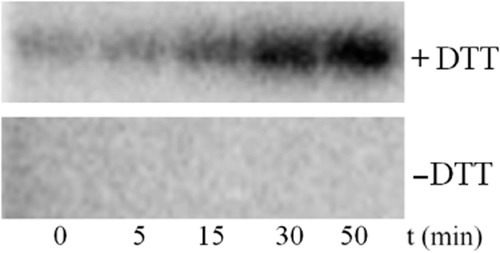
The effect of DTT on TodS autophosphorylation. Autophosphorylation assays of TodS in the absence and presence of 2 mM DTT. TodS (6.5 μM) were incubated with 200 μM ATP containing 4 μCi [γ^32^P] ATP and 0.1 mM toluene. Samples were taken at the time intervals and submitted to SDS-PAGE.

Initial experiments were aimed at determining whether TodS autophosphorylation reducing conditions for activity. Full-length TodS was overproduced in *E. coli* and purified from the soluble fraction of the bacterial lysate. Protein was dialysed against buffer with and without dithiothreitol (DTT) and then submitted to autophosphorylation assays. To this end, TodS was incubated with [γ^32^P] ATP and samples were taken at different times for SDS-PAGE analysis. In the presence of DTT, TodS autophosphorylation activity was observed in agreement with previous studies (Lacal *et al*., 2006; Busch *et al*., 2009) (Fig. 2). However, in the absence of DTT, no activity was observed (Fig. 2) indicating that reducing conditions are essential for activity. Therefore, all subsequent experiments were conducted in the presence of DTT.

### Menadione reduces TodS autophosphorylation

**Fig 3:**
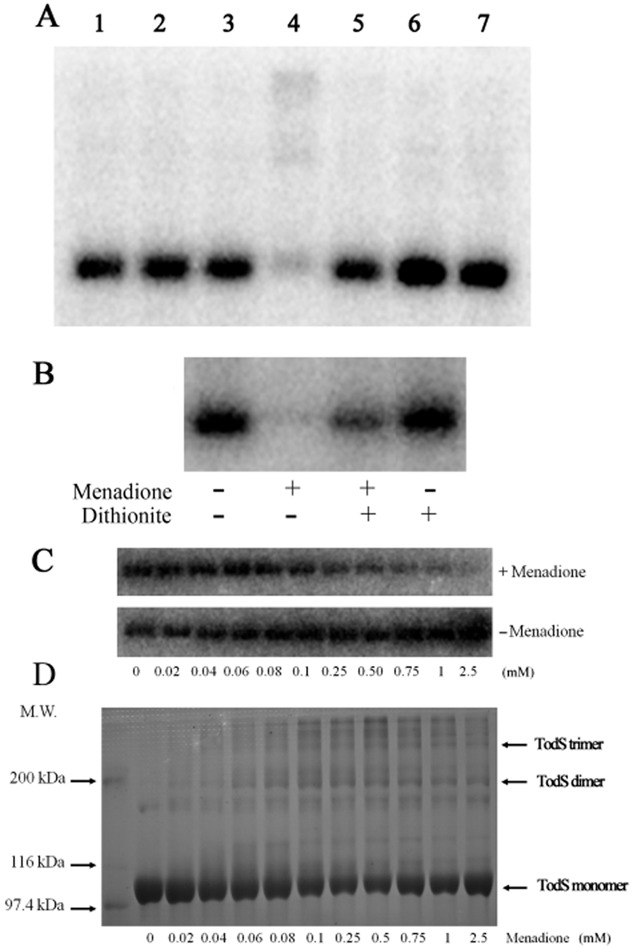
Reduction of TodS autokinase activity by menadione.A. TodS samples in 2 mM DTT-containing analysis buffer were incubated with different compounds at 0.75 mM and then submitted to autophosphorylation assays. Lane 1: buffer, lane 2: H_2_O_2_, lane 3: 20% (v/v) ethanol (control for lane 4), lane 4: menadione, lane 5: duroquinone, lane 6: 3.75% (v/v) chloroform (control for lane 7), lane 7: ubiquinone 10.B. TodS in 2 mM DTT-containing analysis buffer was pre-incubated in buffer, buffer + 0.75 mM menadione, buffer + 0.75 mM menadione and 5 mM dithionite or buffer + 5 mM dithionite for 10 min and submitted to autophosphorylation assays.C. TodS in 2 mM DTT-containing analysis buffer was incubated with 200 μM ATP containing 4 μCi [γ^32^P] ATP in the presence of 0.1 mM toluene and different menadione concentrations. Samples were taken at 30 min and then submitted to an SDS-PAGE.D. TodS in 2 mM DTT-containing analysis buffer was incubated with increasing menadione concentrations and submitted to SDS-PAGE analysis and Coomassie stained.

Subsequently, the influence of the oxidative agents hydrogen peroxide, menadione, duroquinone and ubiquinone-10 on TodS autophosphorylation was assessed. These compounds were chosen because they were found to modulate the activity of other sensor kinases (Bock and Gross, 2002; Swem *et al*., 2003; Malpica *et al*., 2004; Kim *et al*., 2008). As shown in Fig. 3A, no significant changes in TodS autophosphorylation were observed in the presence of hydrogen peroxide, duroquinone and ubiquinone-10. In contrast, TodS autokinase activity was dramatically reduced in the presence of menadione (Fig. 3A). The activity of ArcB was also found to be reduced by menadione (Georgellis *et al*., 2001). The authors showed that menadiol, generated by the reduction of menadione by dithionite, did not cause this reduction (Georgellis *et al*., 2001). Using an analogous approach, TodS was incubated with buffer, menadione, dithionite or a mixture of both compounds (Fig. 3B). In analogy to ArcB, dithionite reduced the magnitude of protein inactivation, indicating that TodS inactivation is due to the oxidizing potential of menadione. To determine the dose–response relationship, TodS activity was measured in the presence of different menadione concentrations (Fig. 3C). Because of the poor solubility of menadione, it had to be added as ethanolic solution. The corresponding amount of ethanol was added to the control where it slightly stimulated TodS activity (Fig. 3C). A menadione concentration-dependent decrease in TodS autophosphorylation was observed (Fig. 3C), and the densitometric analysis of these data resulted in an EC_50_ (concentration at half maximal activity) of 170 ± 25 μM.

To determine whether the effect of menadione is due to a covalent or non-covalent interaction, menadione-treated TodS was dialysed exhaustively into buffer containing 10 mM DTT. As a control experiment, the exhaustive dialysis of untreated protein did not reduce protein activity. However, autokinase activity of menadione-treated TodS did not recover after dialysis, suggesting that menadione causes a covalent TodS modification and that this modification cannot be reversed by treatment with reducing agents.

To elucidate whether menadione causes cross-linking of TodS monomers, the protein was analysed by SDS-PAGE (Fig. 3D). In the absence of menadione, TodS migrated primarily as a monomer, whereas with increasing menadione concentrations, several higher molecular weight species appeared. However, at 2.5 mM menadione, a concentration at which no autokinase was detected, the monomeric form of TodS was largely predominant. This shows that menadione-mediated inactivation is not caused by covalent protein oligomerisation.

**Fig 4:**
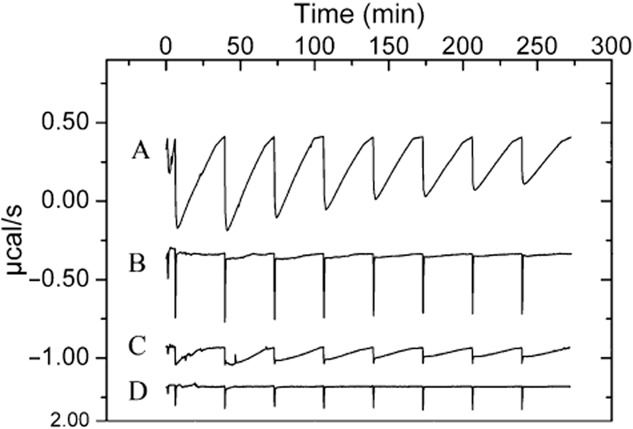
Microcalorimetric titration of TodS with menadione and duroquinone. Heat changes for the injection of a single 1.6 μl and a series of 3.2 μl aliquots of 500 μM menadione or duroquinone into 10 μM TodS or buffer. Analyses were carried out in 2 mM DTT-containing analysis buffer.A. Titration of TodS with menadione.B. Titration of TodS with duroquinone.C. Titration of buffer with menadione.D. Titration of buffer with duroquinone. Traces were set off arbitrarily on the *y*-axis.

The menadione-induced reaction with TodS has also been visualized by isothermal titration calorimetry (Krell, 2008) (Fig. 4). The titration of TodS with menadione gave rise to large exothermic heat signals. Peaks were much broader at its base than peaks observed for binding reactions, indicating that the addition of menadione induced a chemical reaction. This notion is supported by the estimation of the enthalpy change associated with this titration (approximately 2000 kcal mol^–1^), which is largely superior to enthalpy changes caused by binding, which are typically in the range of 5–30 kcal mol^–1^ (Krell, 2008). In contrast, protein titration with duroquinone resulted in narrow peaks that were similar to the buffer control and that are thus due to dilution effects. This is in agreement with the above demonstration that duroquinone does not alter TodS activity.

### Menadione reduces the TodS/TodT mediated upregulation of the P_tod_*_X_* promoter

**Fig 5:**
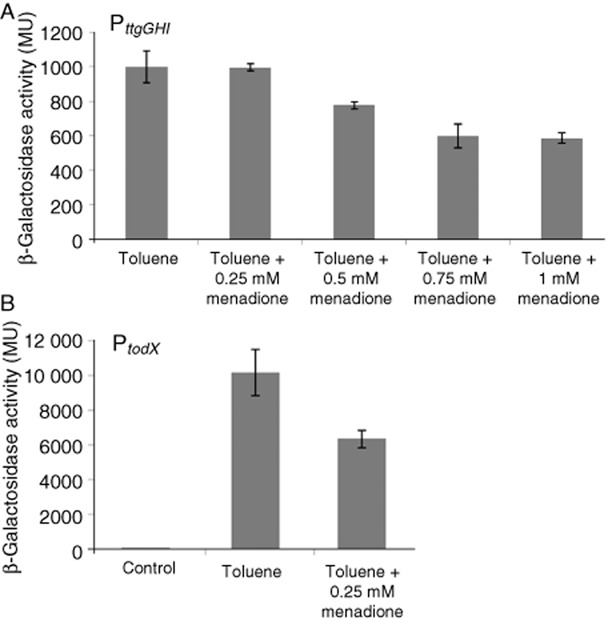
Expression from P*_ttgGHI_* and P*_todX_* in response to menadione.A. Estimation of the pleiotropic effect of menadione. *Pseudomonas putida* DOT-T1E bearing pANA96 (P*_ttgG_*::’*lacZ*) (Rojas *et al*., 2003) was grown on LB medium with 1 mM of toluene in the presence of increasing menadione concentrations.B. Effect of menadione on the expression form P*_todX_*. *Pseudomonas putida* DOT-T1E *ΔtodST* bearing pMIR66 (containing *todST*) and pMIR77 (P*_todX_::′lacZ*) was grown in LB medium with 1 mM toluene in the absence or presence of 0.25 mM menadione. At an OD_660_ = 0.8, β-galactosidase activities were determined.

We have then studied the influence of menadione on P*_todX_* promoter activity. Initial experiments were aimed at determining the pleiotropic effect of menadione on gene expression. As a control, we have used the P*_ttgG_* promoter that has been extensively studied in our group. It is controlled by the TtgV repressor but not by the TodS/TodT system (Rojas *et al*., 2003; Guazzaroni *et al*., 2004; 2005). In analogy to the TodS/TodT system, TtgV responds to different hydrocarbons like toluene (Guazzaroni *et al*., 2005). Using a transcriptional fusion of promoter P*_ttgG_* to the *lacZ* gene, we have assessed the effect of toluene and menadione on gene expression. The β-galactosidase activity in the presence of toluene and absence of menadione was of approximately 1000 Miller units (Fig. 5A), and a similar activity was observed in the presence of 0.25 mM menadione. A reduction in expression at menadione concentrations starting from 0.5 mM was observed. We have therefore studied P*_todX_* expression at a menadione concentration of 0.25 mM. As shown in Fig. 5B, literally no expression from P*_todX_* occurs in the absence of toluene whereas around 10 000 Miller units were observed in its presence. However, in the presence of menadione, the toluene-mediated upregulation of P*_todX_* activity was reduced by around one third (Fig. 5B). This demonstrates that the inhibitory effect of menadione is also observed *in vivo*.

### TodS does not bind cofactors involved in redox sensing

Different molecular mechanisms have evolved for redox sensing, like cofactor containing sensor domains, metal-sulfur clusters and cysteine-based redox sensing (Bauer *et al*., 1999; Zheng and Storz, 2000; Antelmann and Helmann, 2011). TodS was submitted to microcalorimetric titrations with cofactors that were shown in other systems to be involved in redox sensing such as FAD, FMN, heme, NAD, NADH, NADP and NADPH. In all cases, an absence of binding was noted. In addition, TodS sequence analysis showed an absence of sequence motifs typical of metal-sulfur clusters. We therefore hypothesized that menadione-mediated TodS inactivation is based on a cysteine-dependent mechanism.

### Cysteine residues are essential for TodS activity

**Fig 6:**
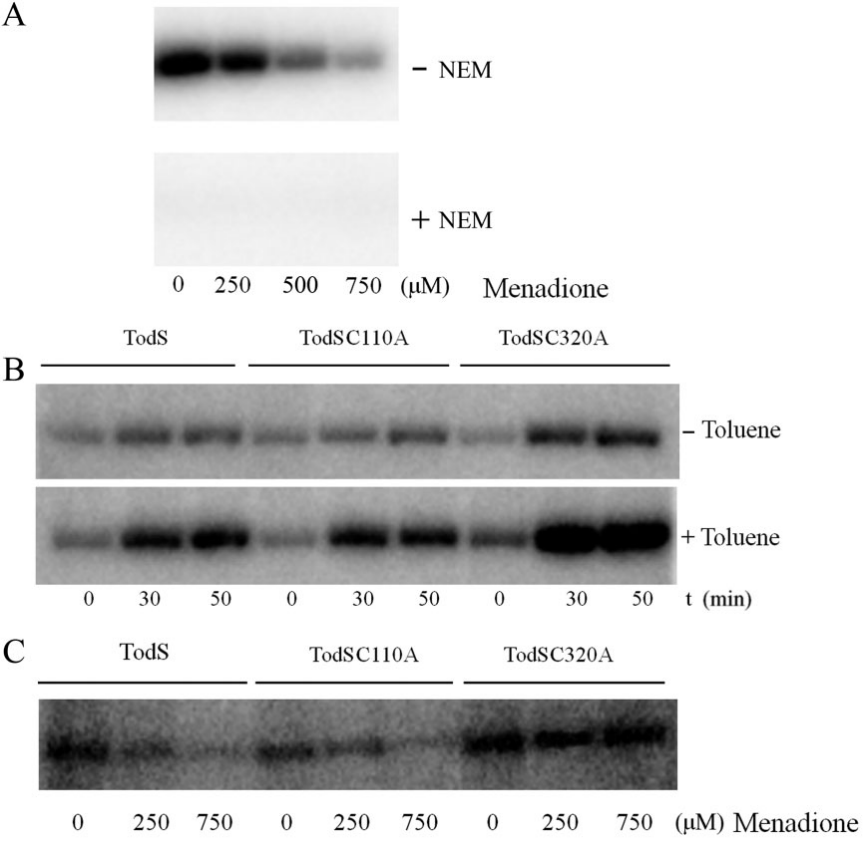
Essential role of cysteines in the menadione-mediated regulation of TodS.A. Effect of alkylation on the kinase activity of TodS. TodS in the 2 mM DTT-containing analysis buffer was incubated with 1 mM NEM for 30 min prior to autophosphorylation assays in the presence of 0.1 mM toluene and different menadione concentrations.B. Autophosphorylation of TodS, TodSC110A and TodSC320A in the absence and presence of toluene. TodS (6.5 μM) were submitted to autophosphorylation assays in the absence and presence of 0.1 mM toluene.C. Effect of menadione on the autophosphorylation of TodS, TodSC110A and TodSC320A. Assays were carried out in 2 mM DTT- and 0.1 mM toluene-containing analysis buffer and different menadione concentrations.

Subsequent experiments were aimed at determining the role of cysteine residues in the menadione-mediated inhibition. TodS was treated with 1 mM N-ethylmaleimide (NEM), a reagent that modifies sulfhydryl groups with high specificity (Smyth *et al*., 1964; Paulech *et al*., 2013). Whereas NEM-untreated protein showed the expected menadione-dependent reduction in autophosphorylation (Fig. 6A), NEM-treated protein was inactive in the absence and presence of menadione, suggesting that cysteines play a central role in TodS activity.

To identify potential cysteines that may be involved in the menadione sensing, the sequences of TodS homologues were aligned (Supporting Information Fig. S1). These proteins were reported or predicted to regulate degradation routes of aromatic compounds (Parales *et al*., 2008; Silva-Jimenez *et al*., 2012). C320 was the only of the 14 TodS cysteines that was entirely conserved in this alignment, whereas C110 was conserved to 75%. C110 is part of the N-terminal PAS domain whereas C320 is located in the catalytic domain of the N-terminal autokinase module (Fig. 1). To assess the role of both amino acids alanine substitution, mutants were generated.

### The autokinase activity of TodS mutant C320A is not modulated by menadione

Purified mutant protein along with wild-type TodS was submitted to autophosphorylation assays. As shown in Fig. 6B, the basal activity of TodSC110A in the absence of toluene is comparable with that of the wild-type protein. However, the basal activity of C320A was found to be around twofold higher than that of wild-type TodS (Fig. 6B). The magnitude of toluene-mediated stimulation of TodS and TodSC110A autophosphorylation activity was also comparable (Fig. 6B). In contrast, phosphorylation of TodSC320A in the presence of toluene was around four times higher than that observed for wild-type TodS (Fig. 6B).

Subsequently, the influence of menadione on protein activity was assessed (Fig. 6C). Menadione had similar effects on TodS and TodSC110A where almost no activity was observed at a concentration of 0.75 mM. In marked contrast, menadione did not cause a reduction of the TodS phosphorylation state in TodSC320A. Taken together, data show that C320 is essential for TodS activity because, firstly, its mutation increases basal TodS phosphorylation, secondly, increases the magnitude of toluene-mediated upregulation of phosphorylation and, most importantly, renders the protein insensitive to menadione. To predict the location of C320 in the structure of TodS a homology model of the TodS fragment 162–406 harbouring the N-terminal autokinase module was prepared (Supporting Information Fig. S2). In this model, C320 is located on a surface-exposed β-strand at significant distance to the ATP-binding site and is therefore unlikely to interfere with nucleotide binding.

### Transcriptional activation by TodSC320/TodT is less affected by menadione than its parental system

**Fig 7:**
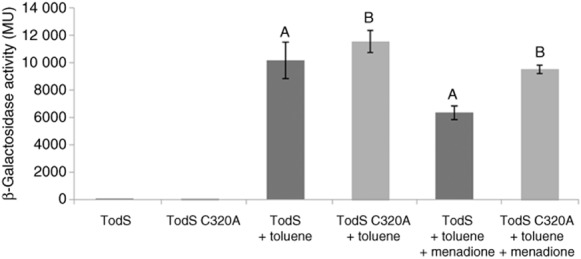
Expression from P*_todX_* mediated by TodS/TodT and TodSC320A/TodT in the absence and presence of menadione. *Pseudomonas putida* DOT-T1E *ΔtodST* bearing pMIR77 (P*_todX_::′lacZ*) and pMIR66 (containing the *todS* and *todT* genes) or pMIR66-C320A (containing *todSC320* and *todT*) were grown in LB medium without toluene, in the presence of 1 mM toluene or in the presence of 0.25 mM menadione and 1 mM toluene. At an OD_660_ = 0.8, β-galactosidase activity was determined.A. *P < 0.05 in Student's *t*-test (*n* = 3), indicating statistical difference.B. *P* > 0.05 in Student's *t*-test (*n* = 3), indicating the results are statistically not different.

Gene expression experiments with a *todS*C320A mutant were conducted. To this end, a pMIR66 derivative (Table 1) was constructed in which the *todS* gene was replaced by the mutant allele. As for the wild-type protein, transcription mediated by the TodSC320/TodT system in the absence of toluene is close to zero. In the presence of toluene, transcriptional activity of the mutant system was slightly above that of the wild type (Fig. 7), which is in agreement with the higher autokinase activity of the mutant system observed *in vitro* (Fig. 6B). Menadione caused the expected reduction in transcriptional activity of TodS/TodT system (*P < 0.05 in Student's *t*-test, *n* = 3, indicative of statistical difference). Menadione also caused a reduction in the mutant system, which however was less pronounced as compared with that of the wild-type system (Fig. 7). When the Student's *t*-test was applied to these results, a *P*-value superior to 0.05 (*n* = 3) was obtained indicating that the transcriptional activity of the mutant system in the presence and absence of menadione is statistically not different. This is consistent with the observation that menadione had no significant effect of the autophosphorylation of TodSC320A (Fig. 6C). However, this slight reduction does not exclude that an alternative mechanism may exist that reduces the activity of the TodSC320A/TodT system *in vivo*.

**Table 1:** Bacterial strains and plasmids used

Strain/plasmid	Relevant characteristics	Reference
Strains		
*Escherichia coli* BL21 (DE3)	F^−^, *ompI, hsdSB* (r ^−^_B_ m^−^_B_)	Studier and Moffatt (1986)
*Pseudomonas putida* DOT-T1E	Tol^+^, wild type	Ramos and colleagues (1995)
*Pseudomonas putida* DOT-T1EΔ*todST*	DOT-T1E, *todST*::Km, Tol^−^	Ramos-Gonzalez and colleagues (2002)
Plasmids		
pMIR66	Gm^R^, containing the *todST* genes	Ramos-Gonzalez and colleagues (2002)
pMIR66-C320A	pMIR66 derivative, containing *todS*C320A mutant gene instead of *todS*	This work
pMIR77	Tc^R^, *P_todX_::′lacZ*	Ramos-Gonzalez and colleagues (2002)
pET28b	Protein expression plasmid	Novagen
pET28b-C110A	pET28b derivative containing *todS*C110A	This work
pET28b-C320A	pET28b derivative containing *todS*C320A	This work
pTodS	pET28b derivative containing *todS*	Lacal and colleagues (2006)
pANA96	pMP220 derivative, containing the fusion *P_ttgG_: ′lacZ*	Rojas and colleagues (2003)

### Menadione-mediated reduction dominates over toluene-mediated stimulation of TodS activity

Data so far show that toluene and menadione have counteractive effects on TodS: toluene increases TodS phosphorylation whereas menadione causes its reduction. The molecular mechanisms for these regulatory actions are different because we have established that the action of toluene is based on its binding to the PAS1 domain (Busch *et al*., 2007) whereas the action of menadione can be associated with C320.

To explore the mutual relationship between these opposing mechanisms, autophosphorylation experiments in the presence of both signal molecules were conducted. The choice of signal concentrations was based on the EC_50_ values determined for the modulation of TodS activity by toluene and menadione. We have shown previously that the EC_50_ for toluene is 10 μM (Busch *et al*., 2007), whereas that for menadione was of 170 μM (see above). Two experiments were conducted in which one signal molecule was present at a constant concentration corresponding to 2× its EC_50_ whereas the other signal was present at varying concentrations corresponding to 0.5, 2, 5 and 10× its EC_50_.

**Fig 8:**
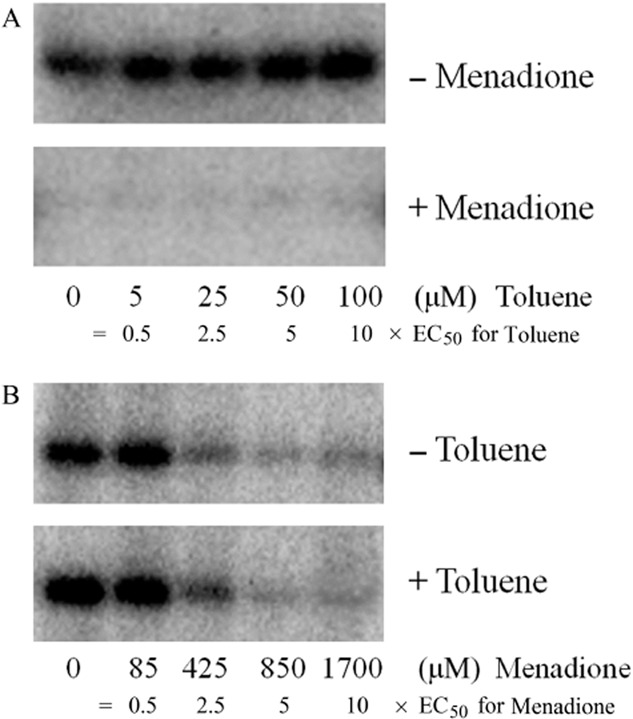
Assessment of the mutual influence of toluene- and menadione-mediated modulation of TodS activity.A. TodS was incubated with 5, 25, 50 and 100 μM toluene (corresponding to 0.5, 2.5, 5 and 10 × EC_50_) in the absence or presence of 340 μM menadione (corresponding to 2 × EC_50_).B. TodS was incubated with 85, 425, 850 and 1700 μM menadione (corresponding to 0.5, 2.5, 5 and 10 × EC_50_) in the absence or presence of 20 μM toluene (corresponding to 2 × EC_50_). Experiments were carried out in 2 mM DTT-containing analysis buffer. Samples were then submitted to autophosphorylation assays.

The first experiment involved the assessment of TodS activity at constant menadione but varying toluene concentrations. Data show that toluene did not stimulate TodS activity in the presence of menadione (Fig. 8A). Subsequently, TodS activity at constant toluene but varying menadione concentration was measured. As shown in Fig. 8B, the addition of menadione to toluene-containing TodS resulted in a dose-dependent reduction of TodS activity, and the magnitude of reduction was comparable with that observed in the absence of toluene. These data demonstrate that menadione-mediated reduction of TodS activity dominates over the toluene-stimulated increase.

## Discussion

The reason for the existence of complex TCS was proposed to lie in the capacity to respond to multiple signals. There are a number of reports that present microbiological and genetic evidence showing that different types of environmental stimuli modulate the action of complex TCSs (Sledjeski and Gottesman, 1996; Kelley and Georgopoulos, 1997; Shiba *et al*., 2004; Geszvain and Visick, 2008). However, in most cases, the nature of the signal as well as the mechanism by which the TCS activity is modulated remains unclear. We have shown previously that TodS activity is regulated by different aromatic hydrocarbons that have either agonistic or antagonistic effects (Lacal *et al*., 2006; Busch *et al*., 2007). The mechanism of action of these signal molecules is based on the non-covalent binding to the TodS PAS1 domain (Fig. 1) (Busch *et al*., 2007).

Here we show that a third type of signal, the oxidizing agent menadione, modulates TodS activity *in vitro* and P*_todX_* expression *in vivo*. There is a number of TCS in which the sensor kinase responds to changes in the redox state by sensing quinone electron carriers like the ArcB/ArcA (Bock and Gross, 2002; Malpica *et al*., 2004), EvgS/EvgA (Itou *et al*., 2009) and BvgS/BvgA (Beier and Gross, 2008; Bekker *et al*., 2010) TCSs. Interestingly, these systems and TodS have in common that they operate by a phosphorelay mechanism (Uhl and Miller, 1996; Perraud *et al*., 2000; Pena-Sandoval *et al*., 2005; Busch *et al*., 2009). ArcB/ArcA, BvgS/BvgA and EvgS/EvgA TCS form a transmitter-receiver-phosphotransfer-receiver phosphorelay, whereas TodS/TodT forms a transmitter-receiver-transmitter-receiver type (Williams and Whitworth, 2010). The reason for this link between the phosphorelay mechanism and their capacity to sense quinone electron carriers remains to be identified.

These above systems differ in their cellular functions. BvgS/BvgA of *Bordetella pertussis* controls the expression of different virulence factors (Beier *et al*., 1995), ArcB/ArcA of *E. coli* mediates gene expression in function of the aerobic/anaerobic state of the bacterium (Malpica *et al*., 2006) and the activation of the EvgS/EvgA causes acid and drug resistance in *E. coli* (Eguchi *et al*., 2011). The demonstration that a TCS controlling a hydrocarbon degradation pathway is also regulated by quinones adds to the functional diversity of redox sensitive hybrid sensor kinases.

The question on the exact mechanism by which TodS and other sensor kinases integrate quinone electron carrier signals is still poorly understood. In this respect, one issue is the subcellular localization of sensing. Quinone signals are located in the membrane, and consequently one may assume that inner membrane sensing kinases detect these signals. Several examples of sensor kinases that recognize their cognate signal within the membrane have been reported (Mascher, 2006; Mascher *et al*., 2006; Cybulski *et al*., 2010). However, TodS and other quinine-sensitive histidine kinases do not appear to sense their ligands in the membrane. ArcB and RegB are anchored to the membrane through transmembrane regions at its N-termini, but these regions appear not be involved in signal sensing. The sensing mechanism of ArcB is based on intersubunit disulfide bond formation involving cysteines 180 and 241, located in the cytosolic PAS domain. A homology model of the ArcB fragment comprising PAS domain and autokinase module (Supporting Information Fig. S3) shows that C180 is in proximity to the membrane whereas C241 is further removed. For RegB, a ubiquinone-binding site has been identified on a periplasmic loop connecting two transmembrane regions (Swem *et al*., 2006; Wu and Bauer, 2010), and a cysteine present on the cytosolic dimerization/phosphotransfer domain was identified as a redox switch that regulates kinase activity in response to aerobic conditions (Wu *et al*., 2013). The exact mechanism by which quinones modulate the activity of ArcB and RegB has not been elucidated.

TodS has no transmembrane regions and is entirely located in the cytosol. However, TodS is not the first cytosolic histidine kinase reported to be sensitive to quinone electron transporters. *In vivo* and *in vitro* experimentation have shown that the activity of the HskA kinase of *P. putida* (Sevilla *et al*., 2013) is modulated by ubiquinone. Apart from their cytosolic location, TodS and HskA share further similarities: (i) both proteins possess two PAS type sensor domains and a receiver domain, which were shown (TodS) (Busch *et al*., 2009) or which may (HskA) form a phosphorelay. (ii) Different algorithms like psortb v3.0 (Yu *et al*., 2010) or pslpred (Matsuda *et al*., 2005) predict that both proteins are located in the cytosol but may be associated with the inner membrane (Sevilla *et al*., 2013). The currently available information therefore suggests that histidine kinases sense quinone electron donors in the vicinity of the membrane. A membrane association of TodS is also in agreement with the hydrocarbon signals that have log partition coefficients octanol/water between 2 and 4 (Ramos *et al*., 2002), indicating a much higher propensity to be present in the organic phase than in the aqueous phase. This property causes these molecules to accumulate within membrane where they reach elevated concentrations (Ramos *et al*., 2002). Therefore, both types of TodS signals, menadione and aromatic hydrocarbons, are primarily present in the membrane and could be sensed by a membrane associated TodS.

A novel aspect of this work concerns the analysis of the mutual influence of the hydrocarbon and menadione-mediated mechanisms (Fig. 8). We show that menadione modulates TodS activity in the presence of toluene, whereas toluene is unable to stimulate TodS autokinase activity in the presence of menadione. The menadione-mediated signalling mechanism dominates thus over the toluene-mediated mechanism. In the case of BvgS/BvgA, it has been established that its activity is modulated by the redox signal ubiquinone in addition to specific ligands that bind to the periplasmic ligand binding domain (Herrou *et al*., 2010). It would be of interest to evaluate potential signal dominance in this and other TCSs.

Several elements indicate that menadione causes a covalent modification of TodS. Firstly, peaks from the microcalorimetric titration of TodS with menadione (Fig. 4) were very broad at its base (typically peaks due to ligand binding only have a width of less than 1 min); secondly, the estimated enthalpy change in isothermal titration calorimetry (ITC) experiments (Fig. 4) is largely superior to enthalpy changes caused by ligand binding; and thirdly, exhaustive dialysis of menadione-inactivated protein did not lead to a recovery of activity. We have generated several peptide maps of active and menadione-inactivated TodS using mass spectrometry. We were able to identify the peptide comprising amino acids 312–329 (harbouring C320) in chymotryptic digests of native TodS and noted its absence from the menadione-modified sample (Supporting Information Fig. S4). We have then searched the peptide maps of menadione-modified TodS for masses corresponding to a number of possible chemical modifications of this peptide (as listed in the legend to Supporting Information Fig. S4), which, however, were unsuccessful in all cases. Therefore, the nature of the chemical modification of C320 could not be established.

What may thus be the physiological reason for the regulation of a hydrocarbon degradation pathway by the oxidative agent menadione? Previous studies have shown that an exposure of *P. putida* to toluene caused a reduction in the transcription of membrane associated enzymes of the respiratory chain leading ultimately to oxidative stress (Dominguez-Cuevas *et al*., 2006) and consequently an enhanced oxidation of menadiol to menadione. The exposure to toluene may have two different effects on *tod* gene expression: (i) a stimulation caused by toluene binding to TodS and (ii) a reduction due to the toluene-mediated generation of oxidative stress signals. The menadione-induced reduction in gene expression may thus represent a negative feedback mechanism caused by the oxidative stress resulting from the presence of organic solvents.

*Pseudomonas putida* can use glucose and toluene for growth, and the use of both carbon sources has been extensively studied. The regulation of both catabolic pathways is tightly interwoven and characterized by simultaneous catabolite repression because glucose inhibits P*_todX_* expression, and toluene was found to affect glucose utilization (del Castillo and Ramos, 2007; Busch *et al*., 2010). Glucose catabolism in *P. putida* occurs through three pathways that converge at the level of 6-phosphogluconate, which is then metabolized by the Edd and Eda Entner/Doudoroff enzymes to central metabolites (Entner and Doudoroff, 1952; del Castillo and Ramos, 2007; del Castillo *et al*., 2007). Interestingly, menadione was found to strongly induce the expression of the operons *zwf/pgl/eda* and *edd*/*glk*/*gltR*2/*gltS* (Park *et al*., 2006; Kim *et al*., 2008). These operons contain genes for glucokinase pathway enzymes (*glk*, *zwf* and *pgl*), a TCS (*gltR2*/*gltS*) for the regulation of the glucose transport system and Entner–Doudoroff pathway enzymes (*edd* and *eda*). The central regulator controlling the expression of both operons is HexR (del Castillo *et al*., 2008; Daddaoua *et al*., 2009). This repressor recognizes and responds specifically to 2-keto-3-deoxy-6-phosphogluconate (KDPG), an intermediate in glucose metabolism and substrate for the *eda* gene product (Daddaoua *et al*., 2009). In the absence of KDPG, HexR is bound at its target promoters, repressing transcription, and KDPG binding triggers protein release and enhances transcription (Daddaoua *et al*., 2009). Most interestingly, HexR was found to be sensitive to menadione, which reduced HexR binding to DNA causing transcriptional activation (Kim *et al*., 2008).

Taken together, striking parallels exists between the central regulators for glucose and toluene metabolism, HexR and TodS/TodT. Both regulators respond to specific signals that correspond to substrates of enzymes involved in glucose and toluene metabolism, KDPG and toluene. These molecules bind to the sensor domains of the corresponding regulators, namely toluene to the PAS1 domain of TodS and KDPG to the sugar isomerase phosphosugar-binding domain of HexR (Lacal *et al*., 2006; Daddaoua *et al*., 2009)*.* In addition, both regulator systems respond to the oxidative stress agent menadione. In the case of TodS, menadione reduces autophosphorylation and gene expression whereas menadione enhances HexR controlled gene expression. Therefore, menadione increases HexR-mediated expression of glucose degradation genes whereas it reduces expression of toluene degradation genes. The opposing effects of menadione on key regulators of toluene and glucose metabolism may be one of the mechanisms responsible for the mutual and simultaneous catabolite repression of *P. putida* grown in toluene and glucose-containing media (del Castillo and Ramos, 2007). In summary, this work provides important insight into the nature of different signals that modulate the activity of complex sensor kinases. Future research will show whether the control of TCS activity by the concerted integration of specific and global signals is a more general feature of hybrid sensor kinases.

## Experimental procedures

### Strains and plasmids

The strains and plasmids used in this study are listed in Table 1.

### Construction of plasmids for protein expression of TodSC110A and TodSC320A

Expression plasmids pET28b-C110A and pET28b-C320A were constructed for the generation of recombinant TodS mutants TodSC110A and TodSC320A. The DNA fragments encoding mutant alleles were generated using an overlapping polymerase chain reaction (PCR) strategy. The initial two PCR reactions were done using pMIR66 as template, which contains the *todS* sequence cloned into the *BamHI* and *NheI* sites of the vector. Forward and reverse primers covering the sequence encoding C110 and C320 and containing the desired mismatch were synthesized (Table 2). The upstream fragments of *todS* were produced using primers PAS1longf and C110Ar or C320Ar. The downstream fragments were produced using primer AK1r and C110Af or C320Af. TodS mutant alleles were produced in a third PCR reaction that contained equimolar amounts of the above products as well as primers PAS1longf and AK1r. The final PCR product was digested with *NdeI* and *Bam*HI, and cloned pET28 (Novagen) linearized with the same enzymes. The resulting plasmids were named pET28b-C110Aor pET28b-C320A. Protein expression from these plasmids gave rise to fusion proteins of TodSC110A or TodSC320A with the N-terminal sequence MGSSHHHHHHSSGLVPRGSH containing the histidine tag.

**Table 2:** Oligonucleotides used in this study

Name	Sequence	Used for construction of plasmid
PAS1f	5′-TAGAACTAGT**GGATCC**	pMIR66-C320A
	TCTAGAGTCG-3′	
C110Ar	5′-TCTCAACATCA*GCG*	pET28b-C110A
	CGAACAAA-3′	
C320Ar	5′-ACTAAGACA*GGC*	pMIR66-C320A
	ACGGATAAG-3′	pET28b-C320A
C110Af	5′-TTTGTTCG*CGC*	pET28b-C110A
	TGATGTTGAGA-3′	
C320Af	5′-CTTATCCGT*GCC*	pMIR66-C320A
	TGTCTTAGT-3′	pET28b-C320A
AK2r	5′-TCAGGAATAC**GCTAGC**	pMIR66-C320A
	GGATGA-3′	
PAS1longf	5′-GTGAGTCATTA**CATATG**	pET28b-C110A
	AGCTCCTTGG-3′	pET28b-C320A
AK1r	5′-AGATGCCC**GGATCC**	pET28b-C110A
	TCATGTG-3′	pET28b-C320A

Restriction sites are highlighted in bold. The mismatch nucleotides are shown in italics.

### Overexpression and purification of TodS, TodSC110A and TodSC320A

*Escherichia coli* BL21 (DE3) was transformed with plasmid pTodS, pET28b-C110A or pET28b-C320A. Cultures were grown in 2 l Erlenmeyer flasks containing 500 ml of Luria–Bertani (LB) medium supplemented with 50 μg ml^–1^ kanamycin at 30°C until an OD_660_ of 0.6, at which point protein production was induced by adding 0.1 mM IPTG. Growth was continued at 16°C overnight prior to cell harvest by centrifugation at 10 000 × *g* for 30 min. Cell pellets were re-suspended in buffer A [20 mM Tris, 0.1 mM ethylenediaminetetraacetic acid (EDTA), 500 mM NaCl, 10 mM imidazole, 5 mM β-mercaptoethanol and 5% (vol/vol) glycerol, pH 8.0] and broken using a French press at 1000 psi. After centrifugation at 20 000 × g for 1 h, the supernatant was loaded onto a 5 ml HisTrap column (Amersham Bioscience), washed with 10 column volumes of buffer A and eluted with an imidazole gradient of 45–500 mM in buffer A. Fractions containing native or mutant TodS were dialysed against analysis buffer 50 mM Tris pH 7.5, 300 mM KCl, 2 mM MgCl_2_, 0.1 mM EDTA and, 10% (vol/vol) glycerol, in the absence or presence of 2 mM DTT, for immediate analysis.

### Plasmid construction for β-galactosidase measurements containing todSC320A

A strategy similar to that described above for the construction of the TodSC320A expression plasmid was used to generate a pMIR66 (Ramos-Gonzalez *et al*., 2002) derivative that contains the *todS*C320A allele instead of the wild-type sequence. In this procedure, the above-mentioned primers PAS1longf and AK1r were substituted respectively by PAS1f and AK2r (Table 2), which contain the *Bam*HI and *Nhe*I cloning sites respectively. These sites permit cloning of the final PCR product into pMIR66 linearized with the same enzymes. All plasmids constructed were verified by DNA sequencing of the insert and flanking regions.

### Autophosphorylation assays under non-reducing and reducing conditions

For analyses under reducing conditions, 6.5 μM of TodS, TodSC110A or TodSC320A in analysis buffer [50 mM Tris-HCl, pH 7.5, 300 mM KCl, 2 mM MgCl_2_, 0.1 mM EDTA, 10% (vol/vol) glycerol and 2 mM DTT] were incubated at 4°C with 200 μM ATP containing 4 μCi [γ^32^P] ATP. For analyses under non-reducing conditions, the same experimental procedures were used except that DTT was omitted from the buffer. Where indicated, TodS or its mutants were pre-incubated in the presence of toluene, NEM, dithionite, menadione, duroquinone, ubiquinone 10 or H_2_O_2_ at the concentrations indicated. At indicated time intervals, samples were taken, the reaction stopped by the addition of 4× SDS sample buffer and then stored at −20°C. After the completion of the time course samples were submitted to SDS-PAGE analysis on 7.5% (wt/vol) SDS-PAGE gels. Protein-associated radioactive phosphorylgroups were visualized on a phosphoimager.

### β-Galactosidase measurements

*Pseudomonas putida* DOT-T1E bearing pANA96 (containing a *P_ttgG_::'lacZ* fusion) was grown overnight in LB medium supplemented with 10 μg ml^–1^ rifampin and 20 μg ml^–1^ tetracycline. Cultures were diluted 100-fold with the same medium, menadione was added at different concentrations and toluene was added at a concentration of 1 mM. When the cultures reached an OD_660_ of 0.8 ± 0.05, β-galactosidase activity was determined in permeabilized cells as described in Ramos-Gonzalez and colleagues (2002). *Pseudomonas putida DOT-T1EΔtodST* bearing pMIR77 (containing a *P_todX_::'lacZ* fusion) and pMIR66 (containing *todST*) or pMIR66-C320A were analysed in parallel using the protocol described above, except that the LB medium was supplemented with 25 μg ml^–1^ kanamycin, 10 μg ml^–1^ tetracycline and 100 μg ml^–1^ gentamycin.

### ITC

ITC experiments were conducted using freshly purified protein and a VP-microcalorimeter (Microcal, Amherst, MA, USA) at 25°C. Protein was dialysed into analysis buffer (50 mM Tris-HCl, 300 mM KCl, 2 mM MgCl_2_, 2 mM DTT, 0.1 mM EDTA, 10% glycerol, pH 7.5) and placed into the sample cell. Three μM TodS was titrated with 500 μM of menadione or duroquinone. For the binding experiments with different cofactors, 10–12 μM TodS was titrated with 250 μM solutions of cofactors. Ligand solutions were prepared in dialysis buffer, and control experiments involved a titration of dialysis buffer with ligand.

## Supporting Information

**Fig. S1.** Sequence alignment of TodS like sensor kinases. The following sequences were used for this alignment: TodS of *Pseudomonas putida* DOT-T1E (Q9EVJ0); TmoS of *P. mendocina* KR1 (Q8KIY1); NodV of *Cupriavidus necator* N-1 (F8GVM0); a homologue from of *Methylibium petroleiphilum* PM1 (A2SDY5); TutC of *Thauera aromatic* (P96139); a homologue of *Dechloromonas aromatica* RCB (Q479E0); StyS of *P. fluorescens* (StyS Pf, O30988); and StyS of *Pseudomonas* sp. Y2 (StyS Ps, O33492). The amino acids that form part of the intra-TodS phosphorelay (Busch *et al*., 2009) are shaded in cyan. Amino acids that are involved in the recognition of hydrocarbons are shaded in green. The conservation of TodS C110 and C320 is highlighted in yellow. The remaining 12 cysteine residues in TodS are shaded in grey. Sequences were aligned using the clustal w multiple sequence alignment algorithm of the NPS@ server (http://npsa-pbil.ibcp.fr/cgi-bin/npsa_automat.pl?page=/NPSA/npsa_server.html). The GONNET protein weight matrix, a gap opening penalty of 10 and a gap extension penalty of 0.2 were used. Fully conserved amino acids are shown in red, highly conserved residues in green and weakly conserved amino acids in blue. **Fig. S2.** The position of C320 in a homology model of TodS comprising amino acids 162–406. This model corresponds to the transmitter module 1 (Fig. 1) and was created using the structure of the entire cytoplasmic portion of a histidine kinase (pdb ID 2C2A) (Marina *et al*., 2005). The model was generated using the CPHmodels 3.2 Server (http://www.cbs.dtu.dk/services/CPHmodels). The phosphorylgroup accepting histidine and C320 are shown in ball-and-stick mode. **Fig. S3.** The cytosolic location of the redox sensing cysteine residues in the ArcB sensor kinase. A homology model of the fragment comprising amino acids 156–504 of the ArcB sensor kinase of *E. coli*. The model was created using the structure of the cytosolic fragment of the ThkA sensor kinase (pdb ID **3A0R)** (Yamada *et al*., 2009) and the Geno3D server (http://geno3d-pbil.ibcp.fr/cgi-bin/geno3d_automat.pl?page=/GENO3D/geno3d_home.html). The cysteines shown by Malpica and colleagues (2006) to be responsible for the integration of the redox signal are shown in ball-and-stick mode. **Fig. S4.** Proteolytic digests of native and menadione-modified TodS followed by liquid chromatography mass spectrometry. A. Sequence of TodS. Shown in red is the chymotryptic peptide of amino acids 312–329 harbouring C320 (underlined). B. High-performance liquid chromatography (HPLC) chromatogram at 214 nm of the chymotryptic digest of TodS. The retention time at which peptide 312–329 (see below) was detected is marked by a red line. C–D. Search within all spectra recorded of the M^+H^ (C) and M^+2H^/2 (D) ions of peptide 312–329 in the digest of native TodS. E. HPLC chromatogram at 214 nm of the chymotryptic digest of menadione-modified TodS. F–G. Search within all spectra recorded of the M^+H^ (F) and M^+2H^/2 (G) ions of unmodified peptide 312–329 in the menadione-modified protein sample. Signals recorded in those scans correspond to noise. The following ions, corresponding to potential modifications of peptide 312–329, were searched for in the peptide maps of menadione-treated TodS: one menadione molecule bound to C320: M^+2H^/2 = 1068.59 and M^+3H^/3 = 712.73; two menadione molecules bound to C320 and C321: M^+2H^/2 = 1155.18 and M^+3H^/3 = 770.45; disulfide bridge between residues C320 and C321: M^+H^ = 1963.0; M^+2H^/2 = 982.5; M^+3H^/3 = 655.0; C320 oxidized to sulfenic acid: M^+H^ = 1980.97; M^+2H^/2 = 990.99; M^+3H^/3 = 660.99; C320 oxidized to sulfinic acid: M^+H^ = 1996.97; M^+2H^/2 = 998.99; M^+3H^/3 = 666.32; C320 oxidized to sulfonic acid: M^+H^ = 2012.97; M^+2H^/2 = 1006.99; M^+3H^/3 = 671.66; all of these searches remained unsuccessful.
