# Supplementary material for: Multiple signals modulate the activity of the complex sensor kinase TodS
Source: Microb Biotechnol. 2014 Jul 1;8(1):103–15. doi: 10.1111/1751-7915.12142 (PMC4321377; doi:10.1111/1751-7915.12142)
Supplement: Supplementary file 1 [file mbt20008-0103-sd1.docx]

**Supplementary Material**

**Multiple signals modulate the activity of the complex sensor kinase TodS**

Hortencia Silva-Jiménez, Álvaro Ortega, Cristina García-Fontana, Juan Luis Ramos and Tino Krell

**Supp. Fig.1. Sequence alignment of TodS like sensor kinases**. The following sequences were used for this alignment: TodS of *Pseudomonas putida* DOT-T1E (Q9EVJ0); TmoS of *P. mendocina* KR1 (Q8KIY1), NodV of *Cupriavidus necator* N-1 (F8GVM0); a homologue from of *Methylibium petroleiphilum* PM1 (A2SDY5); TutC of *Thauera* *aromatic* (P96139); a homologue of *Dechloromonas aromatica* RCB (Q479E0); StyS of *P. fluorescens* (StyS Pf, O30988) and StyS of *Pseudomonas* sp. Y2 (StyS Ps, O33492). The amino acids that form part of the intra-TodS phosphorelay (Busch *et al*., 2009) are shaded in cyan. Amino acids that are involved in the recognition of hydrocarbons are shaded in green. The conservation of TodS C110 and C320 is highlighted in yellow. The remaining 12 cysteine residues in TodS are shaded in grey. Sequences were aligned using the Clustal W multiple sequence alignment algorithm of the NPS@ server (<http://npsa-pbil.ibcp.fr/cgi-bin/npsa_automat.pl?page=/NPSA/npsa_server.html>). The GONNET protein weight matrix, a gap opening penalty of 10 and a gap extension penalty of 0.2 were used. Fully conserved amino acids are shown in red, highly conserved residues in green and weakly conserved amino acids in blue.

10 20 30 40 50 60 70 80

| | | | | | | |

TodS ------------------------------------MSSLDRKKPQNRSKNNYYNICLKEKGSEELTCEEHARIIFDGLY

TmoS ------------------------------------MSSLDKRKTQNRSKKNSYSICLKEKASAELKREELARIIFDGLY

NodV MVYNTSEISGNLQRLQRSVNAGRFLRMIPQRHRNRTMQTINPQLFSHGQTLRVGGVTLRATDGLRTHREKLARIVLDQMY

A2SDY5 ------------------------------------MPNINRQLFAASDSLCVRDVTLRATDDVQTHREKLARIVLDELY

TutC ------------------------------------MTSNNSSVSDISAVLRVRDVTLRAVDDLQTYREKLARVVLDGLY

Q479E0 ------------------------------------MTSEN-AMPNKEMQASVKGVTLVPDDATEVRRQKLARIILDAMY

StyS Ps --------------------------------MPGAWNVMSATDLPGDSVRSVGNVILNPDDSPQTHSEKMARIILDRMY

StyS Pf --------------------------------MPGAWNVMSATDLSGGSVRSVGNVILNPDDSPQTHSEKMARIILDRMY

90 100 110 120 130 140 C110 150 160

| | | | | | | | |

TodS EFVGLLDAHGNVLEVNQVALEGAGITLEEIRGKPFWKARWWQISKKTEATQKRLVETASSGEFVRCDVEILGKSGGREVI

TmoS EFVGLLDAQGNVLEVNQAALNGAGVTLEEIRGKPFWKARWWQISKESVANQKRLVEAASSGEFVRCDIEILGKSGGREVI

NodV QFVGLLDADGLTLEINEAALAGAGIRLDDIQGKPFWEARWWCVSKQTQECAQDAIERACRGEFVRFDVEVYGRTGGEETI

A2SDY5 EFVGLLDAHGTTLEINRAALEGAGIALDDIQGRPFWEARWWATSPEVRREQREVIRRAGEGEFVRRDFEIYGQQGGQETI

TutC EFVGLLDAKGNTLEINQAALDGAGTRLEDIRDKPFWEARWWQVSRETQEEQRKLIARASAGEFVRCDVEIYGRASGEETI

Q479E0 QFLGLLDVDGTVLEINRAALEGAGICLDEVIGKPFWEARWWAISEEARNRVRSMVEQARNGEFVRCDIEIFGDLQGKKSI

StySPs HFAGLLDRDGTILEINLPALEGAGVRIEDIRGTPFWEARWLAVSEESKELQHQLVQRAAAGEFIRCDLEVYGEGSGEQTI

StySPf HFAGLLDRDGTILEINLPALEGAGLRIEDIRGTPFWEARWFAVSQESKALQHQLVQRAAAGEFIRCDLEVYGEGSGEQTI

170 180 190 200 210 220 230 240

| | | | | | | |

TodS AVDFSLLPICNEEGSIVYLLAEGRNITEKKKAEAMLALKNQELEQSVECIRKLDNAKSDFFAKVSHELRTPLSLILGPLE

TmoS AVDFSLLPIRDEQENIVFLLAEGRNITDKKKAEAMLALKNHELEQLVERIRKLDNAKSDFFAKVSHELRTPLSLILGPLE

NodV IVDFSLLPVKDRRNEVMFLLAEARNITEKKRSEAEIVRKNEELQQLLDKIRQIDALKSDFFAKVSHELRTPLALILGPAE

A2SDY5 LIDYSLLPIRDNSGKIVFLLPEGRNITDKKRAEAEIARKNRELQRLLDKIQRLDDAKSDFFANVSHELRTPLALILGPSE

TutC VVDYSILPIRDCNGKVVFLLPEGRNITDKKLAEAELARKNEELQHLLEKIRQLDEAKNEFFANLSHELRTPLSLILGSVE

Q479E0 FVDFSLTPIRDDAGRVAFLLPEGRNITEKIAIEAELTRKNGELQLALEKLREIDGFKTKFFANVSHELRTPLALILGPVD

StySPs VVDYSLTPLRDNHGEVAFLLAEGRNITSKKKYEQEIARKNAELEKLVEQIRMLDEQKNRFFSNLSHELRTPLSLILGPVD

StySPf VTDYSLTPLRDNHGEVAFLLAEGRNITSKKKYEQEIARKNSELEKLVEQIRKLDEQKSRFFSNLSHELRTPLSLILGPVD

250 260 270 280 290 300 310 320

| | | | | | | |

TodS AVMAAEAGRESPYWKQFEVIQRNAMTLLKQVNTLLDLAKMDARQMGLSYRRANLSQLTRTISSNFEGIAQQKSITFDTKL

TmoS TIMEAESGRGSPYWKKFEVIQRNAMTLLKQVNTLLDLAKMDAQQMGLSYRRADLSQLTRVISSNFDGIAQQKSITLDAEL

NodV SLIAGSDNLNEQQRRDLTVIRRNATTLLKHVNDLLDLAKLDAGKISLDYARIDVAHTVRAVAAHFDTLAPQRSYSYVVAL

A2SDY5 SLLATSEGLSDAQRRDLRVIQRNAAMLMKHVNDLLDLAKFDAGKMALRYTRVDLAAEVRTLAAHFEAVAAERSLSYVVQA

TutC SLLADSGDYSGVQRVDLDVIQRNAITLLKYVNDLLDLAKLQAEKLQLHYSRVDLAAVTRMICAHFEALAEYKCLSYVIDA

Q479E0 QMLRESEQLGERERFRLTTIKRNAQSLHQQVNDLLDLARIDAQQMPLAYVCVNVVALLREVAAGFAAAAEERAISLIIEG

StySPs EMLVSS-EFSEHQHTNLASIRRNAVTLLRHVNELLDLAKIDAGKLQLAYELIDITGLVKEITAHFEAHAKQRRIHCAVLS

StySPf EMLVSS-EFSERHDTNLASIRRNAVTLLRHVNELLDLAKVDAGKLQLAYERIDIKGLVEDIAAHFEAHAKQRRIRCAVLS

330 340 350 C320 360 370 380 390 400

| | | | | | | | |

TodS PVQMVAEVDCEKYERIILNLLSNAFKFTPDGGLIRCCLSLSRPNYALVTVSDSGPGIPPALRKEIFERFHQLSQEGQQAT

TmoS PPHLIAEVDCEKYERIILNLLSNAFKFTPDGGLIRCHLSLSQPAHALITVSDSGPGIPQNLRKEIFERFHQLNQEGQQAN

NodV PEACEAEVDPQKFERIVLNLLSNAFKFTPPGGRIRCGLEPSGNSRFLVTVQDSGPGVAPEMRTVLFEPFCQGRAGMAGEF

A2SDY5 PAALEVEVDQQMFERILLNLLSNAFKFTPDFGRIRCSLEANPDHSIQLVVEDSGCGVRADLREEIFERFHQAQSGTTRSF

TutC PAFMEAEVDVEKYERIVLNLLSNAFKFSPDGGRIRCSLSATGTGRILLSIQDSGPGIPADQQSEIFGRFRQGGDIKSRQF

Q479E0 ADELQADVDRAKFARVLANLLSNAFKFTPAGGRICCSITRVANDRFLLSVQDNGPGVPPPMKQQIFDRFAQGQGGLSG--

StySPs PGPILLEADPEKISHVVFNLVANAFNATPDGGRISCHVEIGEGNRCLLTVSDTGPGVPPDMRQRIFERFQQGVEEHGEAR

StySPf PGPILVEADPERIGHVVFNLMANAFNATPDGGRISCRVEIGRGNRCLLTVSDSGPGIPPEMRQRIFERFQQGLEDHGQAR

410 420 430 440 450 460 470 480

| | | | | | | |

TodS RGTGLGLSIVKEFVELHRGTISVSDAPGGGALFQVKLPLNAPEGAYVASNTAPRRDNPQVVDTDEYLLLAPNAENEAEVL

TmoS QGTGLGLSIVKEFVELHHGTISVSDAPGGGALFQVKLPLNAPEGAYVANNAMSRSDNPQTVNPDEYLLPIPTAGSGAELP

NodV GGTGLGLAIVKEFVDLHGGTVSMSEAPGGGALFQVELPLLAPKGTYVRQDVALPQRVVPAVNVDVNEALLATELPYADSR

A2SDY5 SGTGLGLAIAKEFVDLHTGTISVSDAIGGGAQFRVELPSRAPLGAYIRSVDSPLGNRNRGQIVGTIEELQRAEFDAVSDL

TutC GGTGLGLTIVKDFVCLHGGVVVVSDAPGGGALFQIELPRNAPSGVYVNAVAKAGELSPTSFDISAWGLEGRSEWTSAEG-

Q479E0 IGSGLGLNIVKEFVELHFGTVVVLDAPGGGAIFQVEMPKRAPNGVFVRESGEGIG----LVTPQDIDFLEPSSHPASAY-

StySPs AGSGLGLAIVKEFIELHGGTVTVGEAPSSGAIFQVELPAAAPPQVLVRKGSVREQTFSPELPSGGDVSLLPGRGLVSDG-

StySPf AGSGLGLAIVKEFIELHGGTVTVGEAPGSGAIFQVEIPAFAPPQAVVRSGSTGEQAFSHDMSLEADIDIRPGRRLVSDT-

490 500 510 520 530 540 550 560

| | | | | | | |

TodS PFQSDQPRVLIVEDNPDMRGFIKDCLSSDYQVYVAPDGAKALELMSNMPPDLLITDLMMPVMSGDMLVHQVRKKNELSHI

TmoS QFQSDQPRVLIVEDNPDMRCFIRDCLSTDYQVYVAPDGAKALELMCSAPPDLLITDLMMPVMSGDTLVHKVREKNEFAHI

NodV -QALDRPRVLVVEDNVEMGRFLSQILADEYRVEHAMDGSKALAAAIAEPPDLVVTDLMMPELSGDELVAEMRKQASLAQV

A2SDY5 -SGSEKPLVLVAEDNADMRRFIVEVLSSDFRVVHAADGLQALTQARAQAPDAIITDLMMPKLGGDKLVSELRSTPELAHI

TutC --ASDRPRILIVEDNVDMRCFIGRVLIDEYQISVAADGEQALELITSSPPDLVITDLMMPKVSGQLLVKEMRSRGDLANV

Q479E0 --KSGTPRILVVEDNPDLRHFLYDVLIDDYNVTLAANGALALTSALEDPPDLVITDLMMPHFDGEQFVRELRTSGCFPNL

StySPs --RTDLPRVLVVEDNEEMLHLIARTLSNEFSVECASNGKQGFAYMLANPPDLVITDLMLPGMSGEKLIRRMREEGALTQI

StySPf --QADLPRILIVEDNEEMLHLIARTLSSEFSVECAGNGEQGLGLMLANPPDLVIRDLMMPGMSGEKLIRLMREEAQLTQI

570 580 590 600 610 620 630 640

| | | | | | | |

TodS PIMVLSAKSDAELRVKLLSESVQDFLLKPFSAHELRARVSNLVSMKVAGDALRKELSDQGDDIAILTHRLIKSRHRLQQS

TmoS PIMVLSAKPDEKLRVKLLSESVQDYLLKPFSAHELRARVSNLISMKIAGDALRKELSDQSNDIALLTHRLIKSRHRLQQS

NodV PVLVLSARADDALRLELLASSVQDYVIKPFSVHELRVRVRNLIRMKSARDVLQKELASQNDDLGQLARQLIASRQELQRS

A2SDY5 PVLVLSAKADESLRLKLLSDSVQDYIVKPFSSRELLVRVRNIVTMKLAREALQKELASQNEDLAQLTQQLIASKQGLQRS

TutC PILVLSAKADDGLRIKLLAESVQDYVVKPFSATELRARVRNLVTMKRARDALQRALDSQSDDLSQLTRQIIDNRQELQRS

Q479E0 PVLVLSARADDAQRETLLEELVQDYLTKPFSPQELRARVRNLVTVKRTVDILQKELNTQASDVGELTAGLVASRKSLQDS

StySPs PVLVLSARADEELRMTLLATLVQDYVTKPFFIPELLSRVRNLVMTRRARLALQDELKTHNADFVQLARELISGRRAIQRS

StySPf PVLVLSARADEDVRMTLLANMVQDYVTKPFFIPELLSRVRNLVMTRRARLALQDELKTHNADLVQLTRELISGRQAIQRS

650 660 670 680 690 700 710 720

| | | | | | | |

TodS NIALSASEARWKAVYENSAAGIVLTDPENRILNANPAFQRITGYGEKDLEGLSMEQLTPSDESPQIKQRLANLLQGGGAE

TmoS NIALTASEARWKAVYENSAAGIVLTDTENRILNANPAFQRITGYTEKDLAQLSMEQLTPPNERTQMKQRLARLLQSGGAE

NodV LAAERASEQRWRVVFENSAVGIALTDTDGQFMAANPAFRRMLGYTEKELTRLSIESITPAEDRAIARLHIANLVGGKRRE

A2SDY5 HDALKESERRWRAVYENTAVGVSLSDLQGNMHAANPALQEMLGYTESELIGLGNLMTDAEAGHEDRRLQLERLVNGSQVE

TutC HDALQESESRWRAVYENSAAGIVLTNLDGLILSANQAFQKMVGYAEDELRVIEISDLVPEHDREKIRSRVSNLISGRVDD

Q479E0 LVALQISERRWQGLYRNSAVGIALADREGRILKANPALQQMLGYSEAEIVGVSFIDISDESQRAMTLRNVHGLFDGSIDH

StySPs LEAQQKSELRWRAIHENSAVGIAVVDLQWRFVNANPAFCRMLGYTQEELLGHSVLEHTHPDDRNITDQRLHHLLDGRLRT

StySPf LEAQQKSERRWRAIHENSAVGIAVVDLQWRFVNANPAFCRMLGYTQEDVLGYSVLELTHPDDRNITDQRLHHLLDGRLQT

730 740 750 760 770 780 790 800

| | | | | | | |

TodS YSVERSYLCKNGSTIWANASVSLMPQRVGESPIILQIIDDITEKKQAQENLNQLQQQLVYVSRSATMGEFAAYIAHEINQ

TmoS YSVECSYLCKNGSTIWANASVSLMSPRVDEPQVILQIIDDITEKKQAQETLNQLQQELVQVSRSATMGEFAAYIAHEINQ

NodV YRLEKRYSRKDGSAVWVDTSVSLIPGDGSRQSMLVGIVEDITERKRAEHALAQTEAELARVSRVTTMGELAASIAHEVNQ

A2SDY5 MRQQRRYRHRNGMTILANVRESLIPGTSDLPPTLITVVEDITTQKRAEVELAQTKDALARVSRVTTMGELAASIAHEVNQ

TutC YQVQRQCRRKDGRMMWANVRASLIPGLANQSPMVVRIFDDITEKIQTEAELARAREKLTRVMRVTAMGELAASIAHELNQ

Q479E0 YHVQKRYERRDGSFLWANVSASLIPAVDVEGPRLAVIVEDVSSRKEAESALAATQTELARVSRFTAMGELVASIAHEVNQ

StySPs YHHQKRFLHKDGHSLWTRSSVSVIPGSGDTPPLMIGVVEDIDAQKRAEHELERARSELARVMRVTAMGELVASITHELNQ

StySPf YHHQKRFLHKDGHSLWTRSSVSVIPGSGDTPPLMIGVVEDIDEQKRAEHELERARSELARVMRVTAMGELVASITHELNQ

810 820 830 840 850 860 870 880

| | | | | | | |

TodS PLSAIMTNANAGTRWLGNEPSNIPEAKEALARIIRDSDRAAEIIRMVRSFLKRQETVLKPIDLKALVTDTSLILKAPSQN

TmoS PLSAIMTNANAGTRWIGNEPPNIMEAKEALARIIRDSDRAADIIRMVRSFLKRQGPVLKPIDLKALVADTTLILKAPSQS

NodV PLAGVVANGHACLRWLAASPPNEQEAHEAVQRIIRDANRAGNVIARIRQFLKREEPQRTAIRPNEVVSEVISMVQDSLRS

A2SDY5 PLTAVVVNGHACLRWLSTEPRNDLEVQDAIQRIVRDANRASEVIARIRGFLKRSKTDRTMVCMDNVVEDVIGLARDSLRS

TutC PLAAIVTNGHASLRWLGSEPCNLLEAVEAVRRIIHDANRASEIIKRIRGFLQRGEGRRSAVDIFQVVADVAAIVSDMARS

Q479E0 PLSAIVTNSQAALRWLARETPDYQEVVAALNRVNRDASLAGEVIARIRNFLSMGGMQRERLVVRPILENLLQMLQTMLQE

StySPs PLAAMVANSHACRRWLNSSPPNLKEGIASVEAVVRDSQRAAEVVLRLRMFMRRGETQHEPLNLSGVVEEVLGYVRESLVM

StySPf PLAAMVANSHACRRWLNSNPPNLKEGVASVEAVVRDSQRAAEVVLRLRMFMRRGEIQHEPLNLSGVVEEVLDYVRESLVM

890 900 910 920 930 940 950 960

| | | | | | | |

TodS NSVNLDVVADDELPEIWGDGVQIQQLIINLAMNAIEAISQADCETRQLTLSFSGNDTGDALVISVKDTGPGISERQMAQL

TmoS NGVSLNVIAGDTLPAIMGDAVQIQQLVINLAMNSIEAMSQVGCETRQLALSFSSNASNDALIICVKDTGPGIPEDQIGQL

NodV NRISLCEALAPNLPPVAADRVQLQQVILNLVMNAIEAMSLVEGRARVLMVTTQRNDQ-SAVHVAIRDTGVGLDTRQLERV

A2SDY5 AGVQLIKHVDSDLPRVFADSVQLQQVILNLMMNGIEAMGSCATLERQLELRVVKHG--GDIDVSVSDSGTGLVTADFERI

TutC HCIDMRYQAVGQLSLVIADKVQLQQVILNLCINGIESIVGGNSERGELSITVTQSDK-RFLTVSVHDSGPGLAPGEAENV

Q479E0 ADVEVDLRIAPGLPDLLADPVQLQQVLLNLVVNAVDAMREEKERARRLSISVSADTA-GSVLFSVSDTGPGIPPDKAAKI

StySPs QGISLETTLPTDLPMVLADRVQLQQVVLNLVLNAIEAIQAASPSVPRLTLRICRSPDNGPLRLEVEDNGCGVPSSQTERI

StySPf QSISLETTLPTDLPVVLADRVQLQQVVLNLLLNAIEAIQARSPNVPRLKLRICRSPDNGDLQLEVEDNGCGVPALQAERI

970 980 990 1000 1010

| | | | |

TodS FNAFYTTKKEGLGMGLAICLTITEVHNGKIWVECPPAGG-ACFLVSIPARQGSGT----

TmoS FNAFYTTKKEGLGMGLAICLTIAEVHNGKIWAESPPAGG-ACFFVSIPVS---------

NodV FDAFYTTKPQGMGMGLAICRSIVETHGGQLWAMPNDGFG-ASFHFTFPIAASDGS----

A2SDY5 FEAFYTTKPDGMGMGLAICRSIVEAHGGRLWAQANKTQG-LTLQFRLPIAEHAEP----

TutC FDAFYTSKVEGLGMGLAISRSIIEAHGGRLDVLSPSTEGGCTFCFTLPTEEMASPCAPQ

Q479E0 FDALFSTKSRGLGMGLAISRSIVENHGGRLRLVPEAAGG-AHFVFNIPVQP--------

StySPs FEPFYTTKSHGMGMGLAICRTILEAHGGQLNLLPPSDN-CSASGSVFQVILPTDQGTLL

StySPf FEPFYTTKSQGMGMGLAICRTILEAHGGQLNLLPPSDSGSAASGSVFQVVLPTDQGALR

**Supp. Fig. 2. The position of C320 in a homology model of TodS comprising amino acids 162–406.** This model corresponds to the transmitter module 1 (Fig. 1) and was created using the structure of the entire cytoplasmic portion of a histidine kinase (pdb ID 2C2A)([Marina et al., 2005](#_ENREF_2)). The model was generated using the CPHmodels 3.2 Server (ttp://www.cbs.dtu.dk/services/CPHmodels). The phosphorylgroup accepting histidine and C320 are shown in ball-and-stick mode**.**


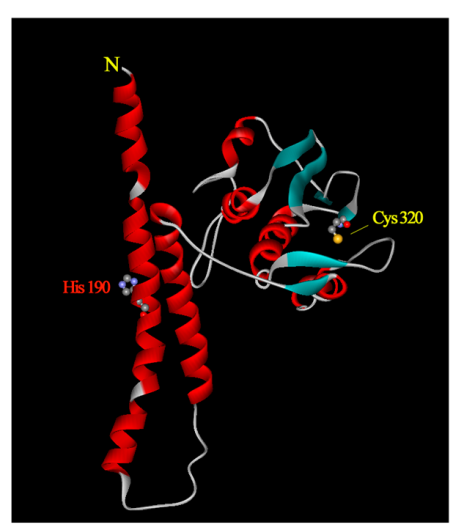


**Supp. Fig. 3.** **The cytosolic location of the redox sensing cysteine residues in the ArcB sensor kinase**. A homology model of the fragment comprising amino acids 156-504 of the ArcB sensor kinase of *E. coli*. The model was created using the structure of the cytosolic fragment of the ThkA sensor kinase (pdb ID **3A0R)** ([Yamada et al., 2009](#_ENREF_3)) and the Geno3D server (http://geno3d-pbil.ibcp.fr/cgi-bin/geno3d_automat.pl?page=/GENO3D/geno3d_home.html). The cysteines shown by Malpica *et al.* ([Malpica et al., 2006](#_ENREF_1)) to be responsible for the integration of the redox signal are shown in ball-and stick mode.


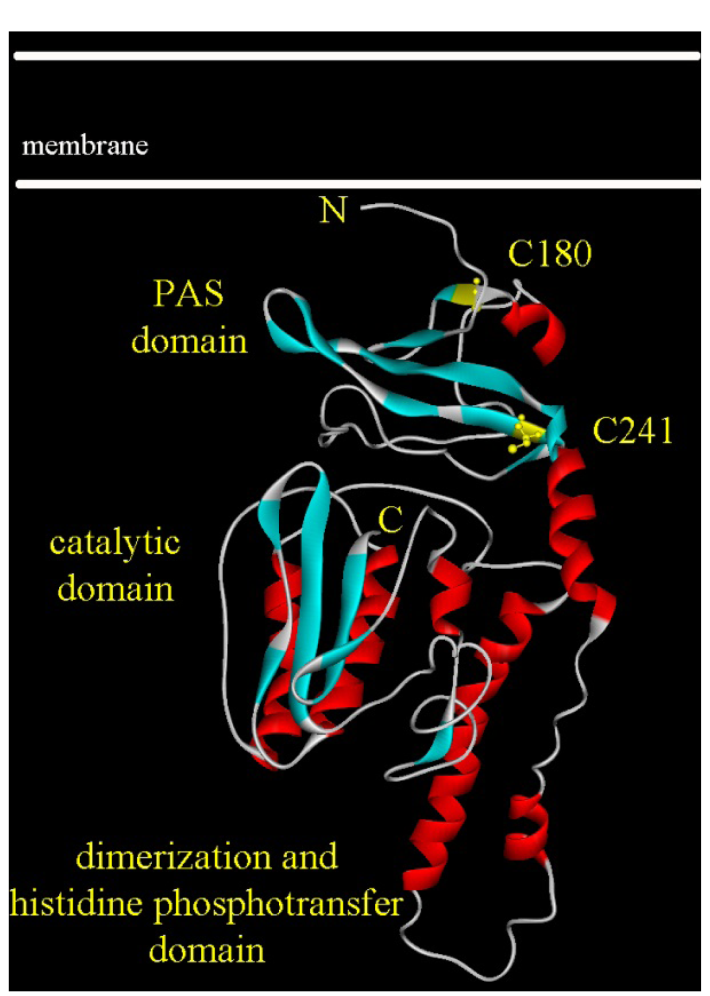


**Supp. Fig. 4) Proteolytic digests of native and menadione modified TodS followed by liquid chromatography mass spectrometry**. A) Sequence of TodS. Shown in red is the chymotryptic peptide of amino acids 312-329 harbouring C320 (underlined). B) HPLC chromatogram at 214 nm of the chymotryptic digest of TodS. The retention time at which peptide 312-329 (see below) was detected is marked by a red line. C-D) Search within all spectra recorded of the M^+H^ (C) and M^+2H^/2 (D) ions of peptide 312-329 in the digest of native TodS. E) HPLC chromatogram at 214 nm of the chymotryptic digest of menadione-modified TodS. F-G) Search within all spectra recorded of the M^+H^ (F) and M^+2H^/2 (G) ions of unmodified peptide 312-329 in the menadione-modified protein sample. Signals recorded in those scans correspond to noise. The following ions, corresponding to potential modifications of peptide 312-329, were searched for in the peptide maps of menadione treated TodS: one menadione molecule bound to C320: M^+2H^/2=1068.59 and M^+3H^/3=712.73; two menadione molecules bound to C320 and C321: M^+2H^/2=1155.18 and M^+3H^/3=770.45; disulfide bridge between residues C320 and C321: M^+H^=1963.0; M^+2H^/2=982.5; M^+3H^/3=655.0.; C320 oxidized to sulfenic acid: M^+H^=1980.97; M^+2H^/2=990.99; M^+3H^/3=660.99.; C320 oxidized to sulfinic acid: M^+H^=1996.97; M^+2H^/2=998.99; M^+3H^/3=666.32.; C320 oxidized to sulfonic acid: M^+H^=2012.97; M^+2H^/2=1006.99; M^+3H^/3=671.66.; All of these searches remained unsuccessful.


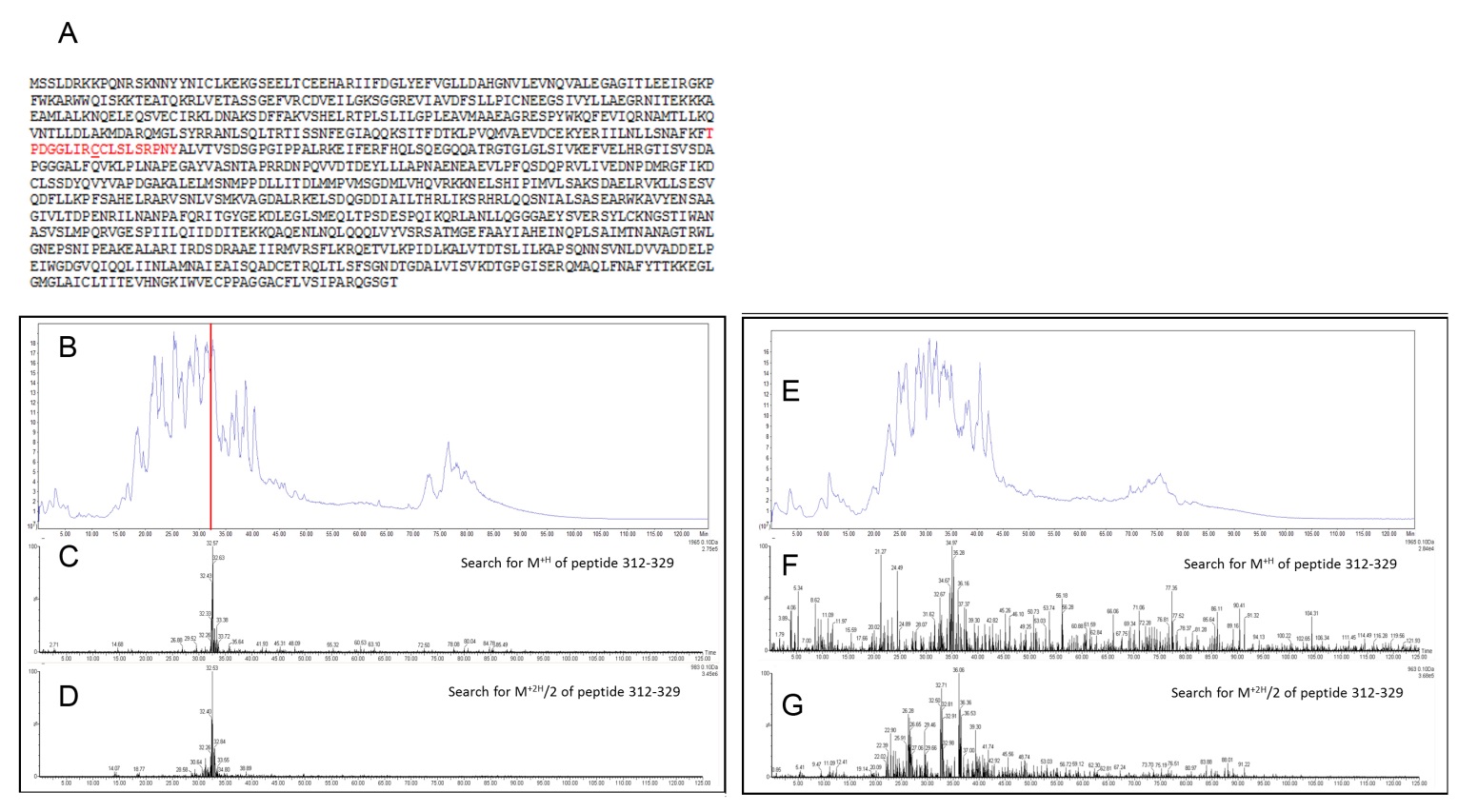


TodS (10 mM) in analysis buffer containing 2 mM DTT was inactivated by a treatment with 2 mM menadione for 60 minutes. Native and menadione treated TodS (10 mM) were digested with 2 % (w/v) chymotrypsin (α-Chymotrypsin from bovine pancreas, Sigma Aldrich C3142) for 45 minutes at 30 ºC. Subsequently another 1 % (w/v) of chymotrypsin was added to the protein and the digestion continued for another 45 minutes. Samples were then frozen at -80 ºC for storage. Peptide separation was carried out on Waters Allience 2695 HPLC equipped with a XSELECT CSH 130 C-18 reverse phase column (Waters). The system was equilibrated in buffer A (2 % acetonitrile/98 % water, 0.1 % (v/v) trifuoroacetic acid) previously degassed with Helium for 2 hours. Digested TodS samples were defrozen and 200 μg of peptides were loaded onto the column. After a washing step in buffer A, peptides were eluted with a linear gradient of buffer A to buffer B (98 % acetonitrile/2 % water, 0.1 % TFA) during 125 minutes at a flow rate of 0.22 ml/min. The column eluent was directly infused into Micromass Quattro microTM API tandem quadrupole system. Spectra were recorded in the positive ion mode at 2 second intervals.

References

Malpica, R., Sandoval, G.R., Rodriguez, C., Franco, B., and Georgellis, D. (2006) Signaling by the arc two-component system provides a link between the redox state of the quinone pool and gene expression. *Antioxid Redox Signal* **8**: 781-795.

Marina, A., Waldburger, C.D., and Hendrickson, W.A. (2005) Structure of the entire cytoplasmic portion of a sensor histidine-kinase protein. *EMBO J* **24**: 4247-4259.

Yamada, S., Sugimoto, H., Kobayashi, M., Ohno, A., Nakamura, H., and Shiro, Y. (2009) Structure of PAS-linked histidine kinase and the response regulator complex. *Structure* **17**: 1333-1344.
